# Negative Correlation between Placental Growth Factor and Endocan-1 in Women with Preeclampsia

**DOI:** 10.1055/s-0038-1670713

**Published:** 2018-10

**Authors:** Marta Ribeiro Hentschke, Edson Vieira da Cunha Filho, Matias Costa Vieira, Letícia Germany Paula, Hiten D. Mistry, Bartira Ercília Pinheiro da Costa, Carlos Eduardo Poli-de-Figueiredo

**Affiliations:** 1 Laboratory of Nephrology, Hospital São Lucas, School of Medicine, Pontifícia Universidade Católica do Rio Grande do Sul, Porto Alegre, RS, Brazil; 2 Division of Child Health, Obstetrics & Gynecology, University of Nottingham, Nottingham, United Kingdom

**Keywords:** pregnancy-induced hypertension, preeclampsia, endothelial function, biomarkers, cytokines, hipertensão induzida pela gravidez, pré-eclâmpsia, função endotelial, biomarcadores, citocinas

## Abstract

**Objective** To analyze endocan-1, a biomarker of vascular endothelial related pathologies, and the placental growth factor (PlGF), an angiogenic factor and a placental dysfunction marker in patients with preeclampsia (PE).

**Methods** Case-control study conducted at Hospital São Lucas, in the city of Porto Alegre, Brazil. Endocan-1 and PlGF levels were quantified in the maternal plasma using the MagPlexTH-C microsphere system (MAGPIX System, Luminex, Austin, Texas, US) and evaluated through analysis of covariance (ANCOVA) and adjusted by body mass index (BMI), gestational age and maternal age. To estimate the difference between the groups, the mean ratio (MR) and the 95% confidence interval (95%CI) were calculated. The Pearson correlation test was used to establish any association between endocan-1 and PlGF levels. The null hypothesis was rejected when *p* < 0.05.

**Results** The group of patients was composed by normotensive (*n* = 67) patients and patients with PE (*n* = 50). A negative correlation between endocan-1 and the PlGF was noted in the entire normotensive group (linear correlation coefficient [r] = −0.605; *p* < 0.001), as well as in the PE group (r = −0.545; *p* < 0.001).

**Conclusion** Endocan-1 levels are increased in patients with PE, and are inversely correlated with PlGF levels. We suggest that it is important to analyze angiogenic and proinflammatory molecules concomitantly in women with PE to better understand the pathophysiology of the disease. Both molecules are strong candidates for PE biomarkers, and future studies will examine any mechanisms connecting these factors in PE.

## Abstract (pt)

**Resumo:**

**Objetivo** Analisar o endocan-1, um biomarcador de patologias vasculares endoteliais, e o fator de crescimento placentário (FCPl), um fator angiogênico, marcador de disfunção placentária em pacientes com pré-eclâmpsia (PE).

**Métodos** Estudo de caso-controle realizado no Hospital São Lucas, em Porto Alegre. Os níveis de endocan-1 e FCPl foram quantificados no plasma materno usando o sistema de microesferas MagPlexTH-C (MAGPIX System, Luminex, Austin, Texas, US) e analisados por análise de covariância (ANCOVA) e ajustados por índice de massa corporal (IMC), idade gestacional e idade materna. Para calcular a diferença entre os grupos, utilizou-se a razão das médias (RM) e o intervalo de confiança de 95% (IC95%). O teste de correlação de Pearson foi utilizado para estabelecer a associação entre os níveis de endocan-1 e FCPl. A hipótese nula foi rejeitada quando *p* < 0,05.

**Resultados** O grupo de pacientes foi composto por pacientes normotensas (*n* = 67) e pacientes com PE (*n* = 50). Uma correlação negativa entre o endocan-1 e o FCPl foi observada em todo o grupo de pacientes normotensas (coeficiente de correlação linear [r] = −0,605; *p* < 0,001), bem como no grupo com PE (r = −0,545; *p* < 0,001).

**Conclusão** Os níveis de endocan-1 estão aumentados em pacientes com PE e inversamente correlacionados com os níveis de FCPl. Sugerimos a importância de analisar moléculas angiogênicas e pró-inflamatórias concomitantemente em mulheres com PE para compreender melhor a fisiopatologia da doença. Ambas as moléculas são fortes candidatos a serem considerados biomarcadores de PE, e trabalhos futuros poderão avaliar quaisquer mecanismos que liguem esses fatores na PE.

## Introduction

Preeclampsia (PE) is one of the 3 major causes of maternal morbidity and mortality in the world, affecting 2 to 8% of all pregnancies.^1^ The etiology of PE remains unknown, but it is thought to begin in placentation, when there is an impairment in the vascular remodeling of the uterine spiral arteries that leads to a decrease in perfusion and high uteroplacental resistance, creating an environment of hypoxia to the placental and fetal tissues. Placental hypoxia results in the release of cytokines that, when exposed to the maternal circulation, change the vascular response, leading to widespread dysfunction of the maternal endothelium.^2^ ^3^ ^4^ ^5^ ^6^ ^7^

Identifying a patient with PE is one of the major goals of prenatal care, so the patient can be referred to high-risk pregnancy protocols, with specific treatment, and, if necessary, plan the termination of the pregnancy.^8^

The endocan-1 molecule is a soluble proteoglycan expressed specifically in endothelial cells. This molecule has been studied in experimental models as well as in vivo, and studies have shown that it is a possible marker and predictor of many diseases^9^ ^10^ ^11^ associated with the vascular endothelium.^9^ ^12^ ^13^ ^14^ ^15^ ^16^ ^17^ ^18^ ^19^ ^20^ ^21^ Therefore, endocan-1 appears to play a key role in tumor progression and in the regulation of the inflammatory process.^22^

Regarding the association of endocan-1 with PE, our group recently published a study that demonstrated a significant increase in endocan-1 levels in the maternal plasma of women with preeclampsia;^23^ subsequently, Chang et al.^24^ demonstrated the same association in the placental tissue, and Cakmak et al^25^ associated higher serum endocan concentrations with the severity of the disease.

A molecule that has been widely associated with the pathophysiology of diseases is the placental growth factor (PlGF). It is produced by the placenta, and has an angiogenic action. During pregnancy, the PlGF is considered a marker of placental dysfunction. Plasma concentrations of PlGF are down-regulated in patients with PE and intrauterine growth restriction (IUGR), and PlGF has been studied as a biomarker and risk predictor for the development of PE.^6^ ^26^ ^27^

In order to predict the chances of developing preeclampsia, it is important to associate molecules that are related both to cell growth and inflammatory cytokines, two key points of PE patients. We hypothesized that there would be a negative correlation between endocan-1 and the PlGF. Thus, the objective of the present study was to correlate the levels of endocan-1 and PlGF in the plasma of pregnant women with and without PE.

## Methods

An observational, case-control study that included pregnant women with a single fetus and with or without diagnosis of PE, who were hospitalized in Hospital São Lucas, Pontífícia Universidade Católica do Rio Grande do Sul (HSL/PUCRS, in the Portuguese acronym), in the city of Porto Alegre, Brazil, between 2010 and 2013. All samples were collected after obtaining informed written consent. The study was approved by the institution's Scientific and Ethics in Research Committee (under no. 11/05352-CEP). Preeclampsia was defined according to the National High Blood Pressure Education Program^28^ and to the VI Brazilian Hypertension Guidelines^29^ as blood pressure ≥ 140/90 mm Hg, associated with pathological proteinuria ≥ 300 mg/24 hours or a proteinuria/creatininuria ratio ≥ 0.3, after 20 weeks of gestation. Early onset PE was considered when the PE developed with gestational age (GA) < 34 weeks. The sample was divided into two groups: one group composed of normotensive (NT) patients, and another group composed of patients with PE. The PE group was called “PE pure” after the pregnant women with superimposed PE and hemolysis, elevated liver enzymes, low platelet count (HELLP ) syndrome were excluded from the analysis.

Data from the maternal identification, the physical examination (upon hospital admission), the previous medical history, the maternal family history, the laboratory tests, the delivery, and the newborn were recorded. For both groups, women were excluded had they had a previous diagnosis of kidney disease, liver disease, active infection, multiple gestation, and/or if there was lack of information in the database.

## Sample Collection

Maternal blood collection was performed after diagnosis (for the PE group) and hospitalization for delivery (for the NT group), in the third trimester of pregnancy. The final sample was composed of 117 patients (50 with PE and 67 NTs). Before delivery, 4 ml of maternal blood were collected in ethylenediaminetetraacetic (EDTA) acid tubes. The samples were processed in the Nephrology Laboratory at HSL/PUCRS, and centrifuged at 2,000 g for 10 minutes, stored in 600 μl aliquots first, at -20°C and then at -80°C until the time of analysis. Laboratory exams to evaluate the severity of the PE were conducted in the PE group.

## Sample Preparation

The samples were prepared according to the instructions of the Milliplex assay kit – MagPlexTH-C assay supplier. To calculate the concentration of molecules, the MagPlexTH-System C – microsphere assay (MAGPIX System, Luminex, Austin, Texas, US), the Milliplex kits HADK2MAG-61K-05 and HCVD1MAG-67K-02 (Millipore Corporation, Billerica, MA, US), and the xPONENT software (Luminex), version 4.2 were used. The intra-assay and inter-assay coefficient of variation was < 10%. The linear correlation coefficient (r) of the standard curve of endocan-1, according to the Luminex instrument, was r = 0.98, and for the PlGF, it was r = 0.99.

### Statistical Analysis

Statistical tests were conducted using the Statistical Package for the Social Sciences (SPSS, IBM Corp. Armonk, NY, US), version 19 for Windows, the Graphpad Prism 6 (GraphPad Software, Inc., San Diego, CA, US) and the WINPEPI (PEPI-for-Windows, © J.H. Abramson, School of Public Health and Community Medicine, Hebrew University, Jerusalem, Israel). The quantitative variables were presented as mean ± standard deviation (SD) or median and interquartile range (IQR) as appropriate, and the Mann-Whitney U-test and the Student *t*-test were used depending on the data distribution. For the categorical variables, we used percentages and applied the Chi-squared test or the Fisher exact test. Correlations between parameters were tested with the Pearson correlation coefficient. The data related to the levels of endocan-1 and PlGF were analyzed by logarithmic transformation by analysis of covariance (ANCOVA) adjusted for body mass index (BMI), GA, and maternal age (presented as a geometric mean). In order to estimate the proportional difference of cytokines between the groups, the mean ratio (MR) and 95% confidence interval (95%CI) were calculated. The magnitude of difference was estimated using the effect size (Cohen d). The null hypothesis was rejected when *p* < 0.05.

## Results

### Study Subjects

The clinical and demographic characteristics, data from the physical examination, the laboratory tests and data collected at the time of delivery are presented in Table 1. The data from the physical examination were collected on the day of admission at HSL/PUCRS.

**Table 1:** Sociodemographic data and maternal and perinatal outcomes from the NT and PE groups

Parameters	NT (n = 67)	PE (n = 50)	*p*-value
Maternal age, years	26 ± 5	26 ± 6.8	0.10
White, n (%)	34 (52)	31 (65)	0.25
Primiparous, n (%)	28 (42)	25 (51)	0.35
Chronic hypertension, n (%)	0 (0)	12 (24.5)	−
Previous PE, n (%)	1 (1.5)	12 (24.0)	−
BMI, kg/m^2^ (weight at end of the pregnancy)	30.4 ± 5.8	32.3 ± 5.4	0.081
SBP, mmHg	119 ± 10	157 ± 17	< 0.001*
DBP, mmHg	75 ± 8	101 ± 14	< 0.001*
GA at delivery, weeks	39.6 ± 1.4	36.7 ± 3.7	< 0.001*
Cesarean section, n (%)	22 (32.8)	38 (76.0)	< 0.001*
5-minute Apgar, n**	9.4 ± 0.6	8.72 ± 1.21	< 0.001*
Birth weight, Kg	3,393 ± 458	2,789 ± 904	< 0.001*
Placental weight, Kg	649 ± 142	590 ± 179	0.063
Hematocrit, %	35.2 ± 2.5	36.22 ± 3.51	0.14
Hemoglobin, g/dL	11.6 ± 0.9	12.31 ± 1.28	0.004*
Platelets, mm^3^ (mil)		211.00 ± 59.05	−
Creatinine, mg/dL		0.81 ± 0.21	−
Proteinuria, P/C rate		0.67 [0.42;2.2]	−
Fasting glucose, mg/dL	75.2 ± 9.3	78.9 ± 13.7	0.26

Abbreviations: BMI, body mass index; DBP, diastolic blood pressure (at admission); GA, gestational age; NT, normotensive pregnancy; P/C, proteinuria/creatininuria; PE, preeclampsia; SBP, systolic blood pressure.

Notes: Data are presented as mean ± SD (Student *t*-test), or absolute numbers and percentages (Fisher exact test), as appropriate. *p < 0.05 for the NT and PE groups. **In the control group, in the fifth minute, no newborn had Apgar index < 7 and in the PE group, 2 newborns received Apgar index < 7.

For the clinical data, we opted to use the GA at delivery data to consider the same period of time for both groups. At the moment of the diagnosis of PE, 21 patients were preterm (10 with GA < 34 weeks) and 20 patients were diagnosed with severe PE due to systolic blood pressure ≥ 160 mm Hg and/or diastolic blood pressure ≥ 110 mm Hg.

### Analysis of the Studied Molecules

The mean levels of PlGF in the NT and PE pure patients were 58.4 pg/mL and 33.05 pg/mL respectively, and the mean levels of endocan-1 were 2032.6 pg/mL and 3357.8 pg/mL respectively. For the statistical analysis, the logarithmic transformation was made, and the ANCOVA was applied. Lower levels of PlGF were found in the PE pure group (MR = 0.38; 95%CI: 0.15–0.95; *p* = 0.041), (Cohen d = 0.54) in the maternal plasma in the PE pure group (MR = 1.56; 95%CI: 1.22 - 2,01; *p* = 0.001) with a moderate effect size.

When the PE group was divided into early PE (< 34 weeks of GA; ≥ 34 weeks of GA and controls), we found in the early PE group lower levels of PlGF (*p* = 0.009) and higher levels of endocan-1 (*p* < 0.001).

**Fig. 1:**
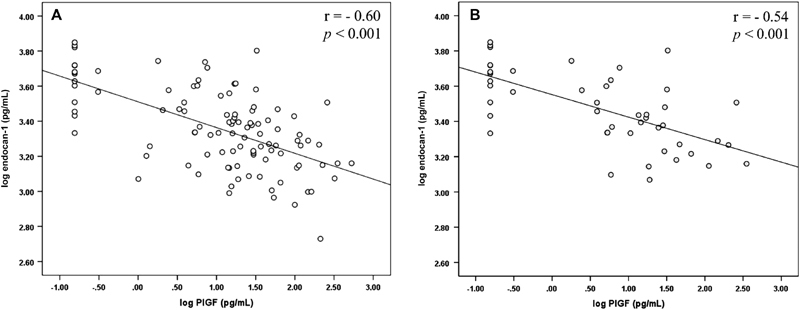
Correlation between endocan-1 and the placental growth factor (PlGF). (**A**) Correlation between endocan-1 and the PlGF in the entire group; (**B**) correlation between endocan-1 and the PlGF in the PE group.

Finally, a negative correlation between endocan-1 and the PlGF was noted in the entire NT group (r = -0,605; *p* < 0.001) and in the PE group (r = –0,545; *p* < 0.001) (Fig. 1).

## Discussion

When compared with the control group, the PlGF was ∼ 60% lower in PE pure patients. In contrast, the level of endocan-1 between the 2 groups was 56% higher in PE pure patients, and a strong negative correlation between the 2 molecules was observed. In the molecular analysis, the early PE group presented a statistically lower level of PlGF and higher level of endocan-1. This could suggest that both molecules may be biomarkers of early onset PE.

Many factors that may be related to PE have been proposed, but the most prominent have been associated with protein receptors of the vascular endothelial growth factor (VEGF) family, particularly the soluble vascular endothelial growth factor receptor-1(sVEGFR1) and the PlGF.^30^ ^31^

The circulating PlGF in human beings is predominantly PlGF-1 (currently there are PlGFs 1 to 4), which is mainly produced by the placenta, and is significantly reduced in cases of PE,^32^ ^33^ due to a negative regulation that occurs under hypoxia,^34^ even before the onset of the PE symptoms.^30^ However, the role of the PlGF in the pathogenesis of PE is not entirely known, partly because its physiologic action is not fully understood. In 2008, however, Osol et al^35^ demonstrated that PlGF-1 is a potent vasodilator, particularly regarding the uterine arteries, and is mediated specially by the release of nitric oxide in pregnancy, which could also regulate the venous tone. In the presence of higher levels of sVEGFR-1, the PlGF is down-regulated, which could diminish the vasodilation process and lead to hypertension, which is observed in patients with PE.^35^

Since the early stages of pregnancy, cell injury occurs in the extracellular matrix and in the vessel walls of the maternal decidua to create a propitious environment for embryo implantation. Ischemic lesions in the placenta resulting from poor remodeling of the decidual vessels release molecular mediators in the maternal circulation, creating an imbalance between vasoconstrictors and vasodilators, culminating in PE syndrome with a progressing systemic response. However, it is thought that in normal pregnancies the syncytiotrophoblast self-renews, leaving apoptotic debris in the maternal circulation, which leads to an expected inflammatory response during placental growth.

The significant increase in the concentration of endocan-1 observed in the maternal plasma might be due to this intense response to this process of physiological development, together with the increased release of proinflammatory cytokines already observed in previous studies.^7^

We questioned which molecule(s) would be mediating the inverse correlation found between the PlGF and endocan-1, and which one seems to change first in the pathophysiology of the disease. There is a lack of studies trying to answer this question clearly. It is known that studies that evaluated both molecules in the first trimester of pregnancy, in separate, showed that both endocan-1 and the PlGF are decreased in patients who developed PE.^36^ Findings from our group demonstrated that the PlGF remains down-regulated, but endocan-1 tends to increase throughout gestation. This occurs, in part, due to the ischemia that begins and compromises the maternal circulation.

All of the patients in our study were included in the third trimester of gestation, and the level of cytokines was adjusted for GA to minimize the influence of this confounding factor in the results.

The placenta plays a crucial role in fetal nutrition. Endocan-1, a cytokine of predominantly inflammatory nature, injures the vasculature, and, therefore, contributes to the reduction in placental flow, IUGR, and to low placental weight.

To our knowledge, there are no complete articles in the literature that correlate these two molecules in PE.

Therefore, the study contributed to previous findings by demonstrating decreased PlGF and increased endocan-1 in the third trimester of pregnancy in PE and its importance in cases of early onset PE. We believe that, in addition to the PlGF, a promising molecule in studies involving PE, endocan-1 also seems to play a role in the PE pathogenesis, and may have a relation with some clinical findings of the disease, but future researches should be performed to clarify these hypotheses.

## Conclusion

The present study evaluated the presence of endocan-1 and PlGF molecules in the maternal plasma; it also correlated the levels of these cytokines in patients with PE and NT patients. In patients with PE, endocan-1 was significantly increased, and the PlGF decreased in the maternal plasma. The role of these cytokines in the pathophysiology of PE needs to be continuously studied.
